# An artificial intelligence-assisted clinical framework to facilitate diagnostics and translational discovery in hematologic neoplasia

**DOI:** 10.1016/j.ebiom.2024.105171

**Published:** 2024-05-28

**Authors:** Ming Tang, Željko Antić, Pedram Fardzadeh, Stefan Pietzsch, Charlotte Schröder, Adrian Eberhardt, Alena van Bömmel, Gabriele Escherich, Winfried Hofmann, Martin A. Horstmann, Thomas Illig, J. Matt McCrary, Jana Lentes, Markus Metzler, Wolfgang Nejdl, Brigitte Schlegelberger, Martin Schrappe, Martin Zimmermann, Karolina Miarka-Walczyk, Agata Patsorczak, Gunnar Cario, Bernhard Y. Renard, Martin Stanulla, Anke Katharina Bergmann

**Affiliations:** a Department of Human Genetics, Hannover Medical School, Hannover, Germany; b L3S Research Centre, Leibniz University Hannover, Germany; c Leibniz Institute on Aging - Fritz Lipmann Institute (FLI), Jena, Germany; d Clinic of Paediatric Haematology and Oncology, University Medical Centre Hamburg-Eppendorf, Hamburg, Germany; e Research Institute Children’s Cancer Centre Hamburg, Hamburg, Germany; f Hannover Unified Bio Bank, Hannover Medical School, Hannover, Germany; g Department of Paediatrics, University Hospital Erlangen, Erlangen, Germany; h Department of Paediatrics, University Medical Centre Schleswig-Holstein, Campus Kiel, Kiel, Germany; i Department of Paediatric Haematology and Oncology, Hannover Medical School, Hannover, Germany; j Department of Paediatrics, Oncology and Haematology, Medical University of Lodz, Lodz, Poland; k Hasso Plattner Institute, Digital Engineering Faculty, University of Potsdam, Potsdam, Germany

**Keywords:** Clinical framework, Data integration, Machine learning, Leukaemia

## Abstract

**Background:**

The increasing volume and intricacy of sequencing data, along with other clinical and diagnostic data, like drug responses and measurable residual disease, creates challenges for efficient clinical comprehension and interpretation. Using paediatric B-cell precursor acute lymphoblastic leukaemia (BCP-ALL) as a use case, we present an artificial intelligence (AI)-assisted clinical framework clinALL that integrates genomic and clinical data into a user-friendly interface to support routine diagnostics and reveal translational insights for hematologic neoplasia.

**Methods:**

We performed targeted RNA sequencing in 1365 cases with haematological neoplasms, primarily paediatric B-cell precursor acute lymphoblastic leukaemia (BCP-ALL) from the AIEOP-BFM ALL study. We carried out fluorescence in situ hybridization (FISH), karyotyping and arrayCGH as part of the routine diagnostics. The analysis results of these assays as well as additional clinical information were integrated into an interactive web interface using Bokeh, where the main graph is based on Uniform Manifold Approximation and Projection (UMAP) analysis of the gene expression data. At the backend of the clinALL, we built both shallow machine learning models and a deep neural network using Scikit-learn and PyTorch respectively.

**Findings:**

By applying clinALL, 78% of undetermined patients under the current diagnostic protocol were stratified, and ambiguous cases were investigated. Translational insights were discovered, including *IKZF1*^plus^ status dependent subpopulations of *BCR::ABL1* positive patients, and a subpopulation within *ETV6::RUNX1* positive patients that has a high relapse frequency. Our best machine learning models, LDA and PASNET-like neural network models, achieve F1 scores above 97% in predicting patients’ subgroups.

**Interpretation:**

An AI-assisted clinical framework that integrates both genomic and clinical data can take full advantage of the available data, improve point-of-care decision-making and reveal clinically relevant insights promptly. Such a lightweight and easily transferable framework works for both whole transcriptome data as well as the cost-effective targeted RNA-seq, enabling efficient and equitable delivery of personalized medicine in small clinics in developing countries.

**Funding:**

German Ministry of Education and Research (BMBF), 10.13039/501100001659German Research Foundation (DFG) and 10.13039/501100001870Foundation for Polish Science.

**Research in context**

**Evidence before this study**

We systematically searched PubMed for studies published before 1st of August 2023, which were related to oncological frameworks for data integration and interpretation. We used keywords “clinical frameworks” OR “clinical interpretation” AND “oncology” OR “cancer”. We identified several studies such as Clinical Interpretation of Variants in Cancer (CIViC), and Molecular Oncology Almanac (MOALmanac), and OncoKB. All these previous works, however, were focused on Next Generation Sequencing data, and did not integrate much non-genomic data, such as drug response and measurable residual disease (MRD), which are critical for clinical decision-making. Furthermore, these frameworks were designed for all cancer types which may not bring the full potential for a specific cancer entity. For example, in leukaemia, gene fusions have higher relevance in guiding treatment decisions than point mutations which are often important in solid tumours. We also searched PubMed for studies related to machine learning classifiers in leukaemia. We used the keywords (“machine learning” OR “subtype”) AND “leukaemia” AND (“RNA-seq” OR “gene expression”). We identified previous studies of subtyping leukaemia based on gene expression data. For example, Schultze et al. collected over 12,000 samples and surveyed ten machine learning algorithms to predict the presence of acute myeloid leukaemia (AML). Schmidt et al. developed ALLSorts, a machine learning tool that employs RNA-seq data and feature creation to classify BCP-ALL subtypes. However, those studies in leukaemia used only shallow machine learning models and did not explore deep neural networks. A pathway-associated sparse deep neural network (PASNet) is one of the pioneering works to apply deep NN to RNA-seq data. It was developed to predict long-term survival of glioblastoma. P-NET has advanced this idea even further by incorporating many other types of genomic data and developed an interpretable deep NN that achieved superior predictive performance for treatment resistance in prostate cancer. However, most of these AI applications are proof-of-concept studies using publicly available or retrospective datasets, and are not yet integrated into routine diagnostics to benefit patients in real time.

**Added value of this study**

To our knowledge, clinALL is a key early clinical framework that is integrated into routine diagnostics and bring together diverse genomic and clinical information through a global “second-order” analysis. It presents clinicians and researchers with a comprehensive overview of the data in real time, supporting the point-of-care clinical decision making as well as the discovery of translational insights. clinALL also incorporates a deep neural network into routine diagnostic analyses of cancer genomic data, providing a fast and robust tool for patient subtyping.

**Implications of all the available evidence**

clinALL represents an approach for data integration, visualization and analysis in a diagnostic setting. By using BCP-ALL as a use case, we demonstrated its utility in supporting routine diagnostics as well as translational research. This easily transferable framework works for both whole transcriptome data as well as the cost-effective targeted RNA-seq data. By using clinALL and similar frameworks for other disease entities, personalized medicine can be delivered more efficiently and equitably across the globe.

## Introduction

With the increasing use of next generation sequencing technology (NGS) in oncological practice, we are in urgent need of clinical interpretation frameworks that can integrate and analyse the growing quantity and complexity of data. OncoKB, St. Jude Ecosystem, Clinical Interpretation of Variants in Cancer (CIViC), and Molecular Oncology Almanac (MOALmanac) are pioneering works that demonstrate the significant value of such frameworks.^1^, ^2^, ^3^, ^4^ MOALmanac even takes into account “second order” genomic alterations, a term that refers to either synergistic effects among single gene-variants or global molecular features such as TMB (Tumour Mutation Burden) and global gene expression pattern.^4^ This is in contrast to “first order” relationships where individual genetic aberrations are paired with clinical actions. However, previous frameworks have not considered or integrated non-genomic data critical for clinical decision making, such as drug responses, measurable residual disease (MRD) and immunophenotypes. Furthermore, each cancer entity is distinct, and a generic framework for all cancer types may not bring the full potential for a specific cancer entity. For example, in leukaemia, gene fusions have higher relevance for choosing the best treatment strategy compared to point mutations, which often guide treatment decisions for many solid tumours.^5^ Therefore, we propose that a cancer-specific clinical interpretation framework, which integrates genomic data as well as relevant clinical and diagnostic data, will ultimately improve clinical decision-making and reveal insights for translational research.

A growing number of artificial intelligence (AI) applications are now being developed for clinical settings, mainly in the imaging domain, but recently also in genomics.^6^, ^7^, ^8^ Various machine learning models are used for leukaemia subtyping, and to analyse drug responses and predict survival.^9^, ^10^, ^11^ Further, deep learning neural networks (NN) present a greater capacity to efficiently analyse complex relationships in large genomic datasets towards potentially more impactful clinical outcomes. PASNet and P-NET are such pioneering works and have achieved superior performance in glioblastoma and prostate cancer respectively.^12^^,^^13^ However, most of these AI applications are proof-of-concept studies using publicly available or retrospective datasets, and are not yet fully implemented into routine diagnostics to benefit patients in real time.

BCP-ALL is the most common childhood cancer. Risk-adapted therapy incorporating our understanding of genetic alterations have greatly improved treatment outcomes.^5^^,^^14^ Treatment protocols and risk stratifications vary slightly worldwide, but are all based on genetic aberrations, clinical information such as MRD and drug responses.^5^^,^^15^ Despite significant progress in research, integrating genomic data into routine diagnostics to improve real-time clinical decision-making remains challenging. This is especially true in developing countries where 80% of the childhood acute leukaemia occurs but bioinformatics support is only rarely available.^16^ Here we present clinALL, an AI-assisted data integration and analysis framework that is ready to be used for different hematologic neoplasia in routine diagnostics.

## Methods

### Patient cohort

In total, 1365 patients (760 male and 605 female) from the AIEOP-BFM ALL (n = 1248), EsPhALL (n = 44), Interfant (n = 5) and ALLTogether (n = 46) studies and 22 patients with suspected ALL were included in this study. Median age at the diagnosis was 5 years (range 0–19 years). Patients were selected by availability of targeted RNA-seq data, with appropriate quality (>3 million coverage, 10 split reads). Most of the ALL subgroups were included, but *BCR::ABL1* and *ETV6::RUNX1* subgroups were underrepresented, since the treatment protocol does not include routine RNA-seq for these subgroups. The number of samples used in machine learning is slightly smaller than in Uniform Manifold Approximation and Projection (UMAP) because the latest samples were not included. For the independent datasets used for machine learning model validations, we used 174 whole transcriptome cases from Chouvarine et al., and 503 targeted RNA-seq cases from Urbanska et al.^17^^,^^18^

### Karyotyping, FISH and RNA sequencing

Bone marrow (BM) or peripheral blood (PB) was obtained at the time of initial diagnosis. The tumour cell content was above 40%. DNA was extracted using QIAamp DNA Blood Midi Kit, and the RNA extraction was performed using the RNeasy, or QIAmp RNA Blood Mini Kit (Qiagen, Hilden, Germany, catalogue numbers: 51185, 74106 and 52304, respectively). Karyotyping and FISH analysis were done as previously described.^17^^,^^19^ For targeted RNA-seq, TruSight RNA Pan-Cancer Panel (Illumina, San Diego, USA, catalogue number: RS-303-1002) was performed according to the manufacturer's instructions. The probes in the panel target 1385 cancer genes, and fusions were detected by Illumina pipeline TopHat and RNA-Seq. For the independent dataset from Chouvarine et al., the whole transcriptome RNA sequencing was carried out using Illumina’s TruSeq Stranded Total RNA Prep kit (catalogue number: 20040525).^17^ For the independent dataset from Urbanska et al., targeted RNA-seq was carried out using TruSight RNA Pan-Cancer Panel from Illumina (catalogue number: RS-303-1002). To get the gene expression counts, we used the RNA analysis pipeline from MegaSAP (https://github.com/imgag/megSAP). We normalized the raw counts by VST (variance stabilizing transformation) normalization in PyDeseq2 (v0.3.5) before applying UMAP (v0.5.3) analysis or developing machine learning models.

### Differential gene expression (DGE) analysis and pathway analysis

DGE analysis was done by DESeq2 (v1.36.0). Raw counts were used as input and the volcano plots were generated by EnhancedVolcano (v1.14.0) in R. The pathway analysis was carried out with Reactome pathway analysis tool (v3.7, Reactome database release 84).^20^ We used all the differentially expressed genes that have adjusted p-value less than 0.05 as input.

### clinALL software

clinALL is an open-source python package for data integration, visualization and analysis for haematological neoplasia. It takes inputs of a raw or normalized gene expression file, a label file, clinical and genetic data files in tsv format, and generates an interactive html file. The main graphics displayed in html are based on UMAP (v0.5.3) analysis of the RNA-seq data. We used Bokeh (v2.4.3) to develop the interactive web interface. clinALL trains the final model on all patients with known subgroups, and provides subgroup recommendations for undetermined cases. It incorporated our top two machine learning models. Users have the options of (1) using LDA model or PASNE-like model; (2) using the provided model or train their own model; (3) starting with gene expression raw counts or normalized counts. Our git repository offers two saved models, one LDA and one PASNET-like, both trained on our real data. For demonstration purposes, we include a synthetic dataset containing 1600 samples and 1500 genes, along with the LDA and PASNET-like models trained on this synthetic data. We also provide a script which allows users to generate their own synthetic datasets of arbitrary size.

### Machine learning models

For shallow machine learning models, we used scikit-learn estimators where KNN, LR, LDA and RF are inherently multiclass and SVM is multiclass as one-vs-one. We used VST normalized counts as input and repeated each experiment three times with nested cross validations (5-outer-fold-4-inner-fold). Specifically, we stratified data into five (outer) folds to create outer loop training and testing sets. Then we stratified each training set into four (inner) folds and used each as a validation set for hyperparameter tuning. After finding the best hyperparameters using grid searches, we set the model with this best hyperparameter and apply it to the outer loop test set. For the deep neural network model, we rewrote the PASNet code with the latest version of PyTorch, modified it for multiclass classification, and replaced the sigmoid activation function with ReLu activation because it trains much faster with less chance of vanishing gradient. The gene-pathway network was adapted to our targeted RNA-seq and has fewer nodes in the input gene layer and pathway layer (1068 and 470 nodes respectively). The model training was done with GPU RTX3080. For the independent dataset from Chouvarine et al., we first extracted the expression profile of 1506 genes that also exist in our targeted RNA-seq panel and removed the batch effect by Combat-seq.^21^ Then we trained our models on 80% of our own data, and performed testing on 20% of own data and the independent dataset.

### Ethics

The usage of leukemic samples was approved by the institutional review board of the Medical Faculty of the Christian-Albrechts-University Kiel (AIEOP-BFM ALL 2009: A177/09; INTERFANT 06: A103/08, AIEOP-BFM ALL 2017: A105/18, EsPhALL: A 123/18). The usage of leukemic samples from the ALLTogether trial was reviewed by the institutional board of the ALLTogether trial. The usage of the patient derived data from Urbanska et al. was approved by the Ethics Committee of the Medical University of Lodz. Informed consent was obtained from all participants. Data was managed in accordance with the General Data Protection Regulation.

### Statistics

We did statistical analysis to compare the performance of different machine learning models. First, we made quantile–quantile plot to examine if our data is normally distributed. Then we calculated p-value for two tailed T-test for each of the model pair. P-value smaller than 0.05 is considered statistically significant.

### Role of funders

The funding sources did not play a role in any aspect of manuscript or decision to submit for publication.

## Results

**Fig. 1:**
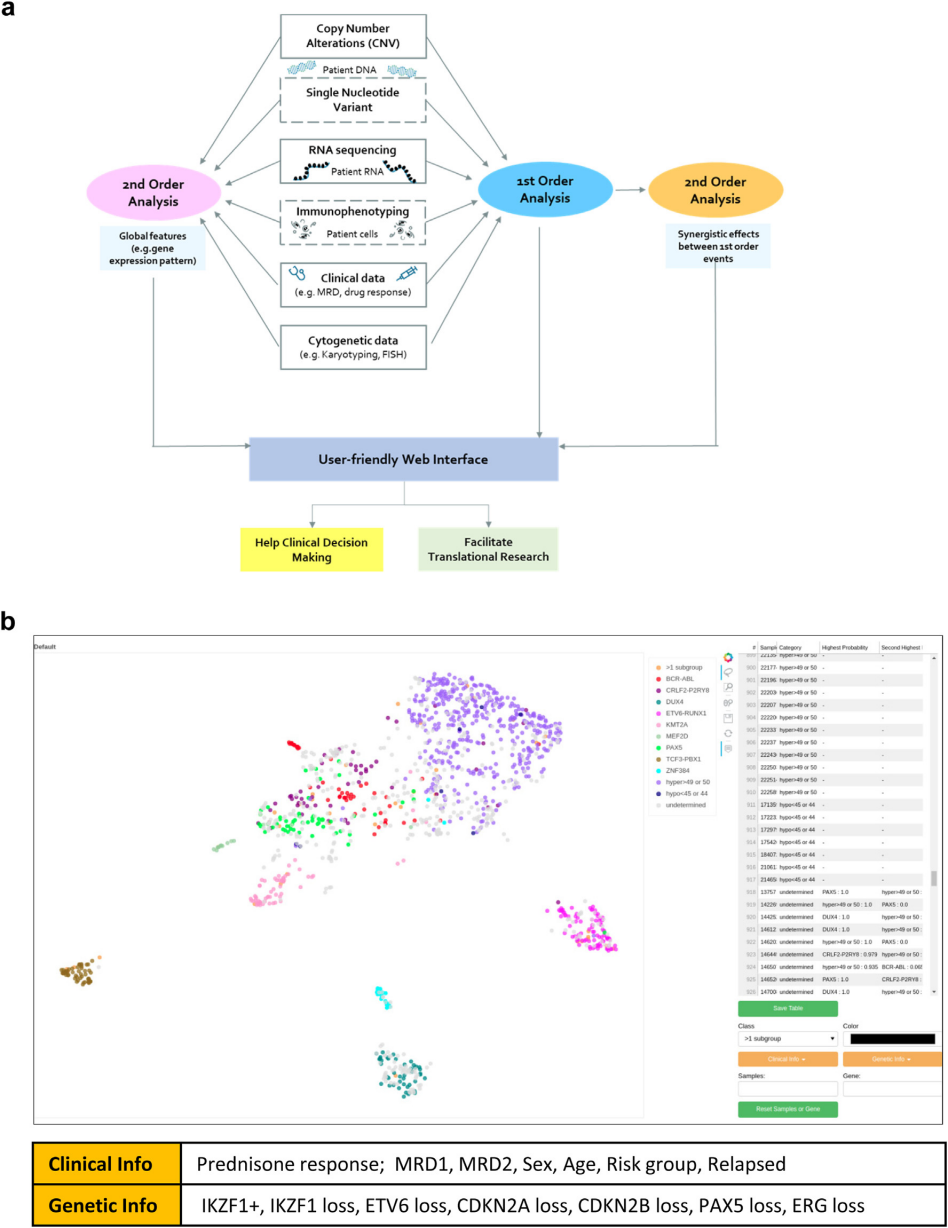
**clinALL, a clinical framework for hematologic neoplasia.** (a) clinALL workflow. The types of data routinely generated in clinics are shown in the boxes in the middle. The dashed boxes indicate the data that has not been integrated yet. clinALL integrates and analyses not only the first order but also second order genetic and clinical information. The second order information could be either the synergistic effects among first order events (orange oval on the right) or global molecular features (pink oval on the left). The analysis results are presented in a user-friendly web interface to support clinical decision making and translational research. (b) A screenshot of the clinALL web interface. The main graph is based on the Uniform Manifold Approximation and Projection (UMAP) analysis of targeted RNA-seq data. Each colour represents a patients’ subgroup that is determined by conventional molecular cytogenetics and fusion calling. The predictions of patients’ subgroups by machine learning are shown in a table on the right side of the interface. The types of clinical and genetic information that can be displayed for BCP-ALL use case are listed at the bottom.

clinALL is a framework that integrates second order genomic information and clinical information to support clinical decision-making. We developed clinALL using population-wide data based on the AIEOP-BFM, EsPhALL, Interfant and ALLTogether studies,^15^^,^^22^, ^23^, ^24^, ^25^ which include nearly all paediatric patients with BCP-ALL in Germany. According to the study protocols, genome data such as those derived from targeted RNA sequencing and copy number alteration analyses are routinely generated in addition to cytogenetic, immunophenotypic, and clinical data as well as MRD and drug response. We used this comprehensive dataset to develop our clinical framework and presented results of the analysis in a user-friendly web interface (Fig. 1a). The main interface is based on Uniform Manifold Approximation and Projection (UMAP) analysis of the RNA sequencing data (Fig. 1b). A range of clinical and genomic information, including a second order genomic information *IKZF1*^plus^ status, can be displayed on top of the UMAP (Supplementary Fig. S1a). Users can not only visualize the UMAP location of individual patients by their unique IDs, as demonstrated in Supplementary Fig. S1b using iAMP21 patients as an example, but also choose any area on the UMAP and receive an AI-generated prediction of the patients’ subgroup.

Flexibility and the ability to generate outputs promptly were the key considerations when designing clinALL. As soon as new patient data arrives, clinALL can create an interactive web interface within a few minutes. We plan to re-train the models only when the sample size increases substantially. The training of the neural network takes less than 1 h on a GPU RTX3080. clinALL was developed on BCP-ALL data, but it is ready to be used for other haematological neoplasia, such as AML and T-ALL etc., as long as RNA sequencing data are generated (Supplementary Fig. S1c). The package is publicly available at https://git.l3s.uni-hannover.de/tang/clinALL with a synthetic dataset.

**Fig. 2:**
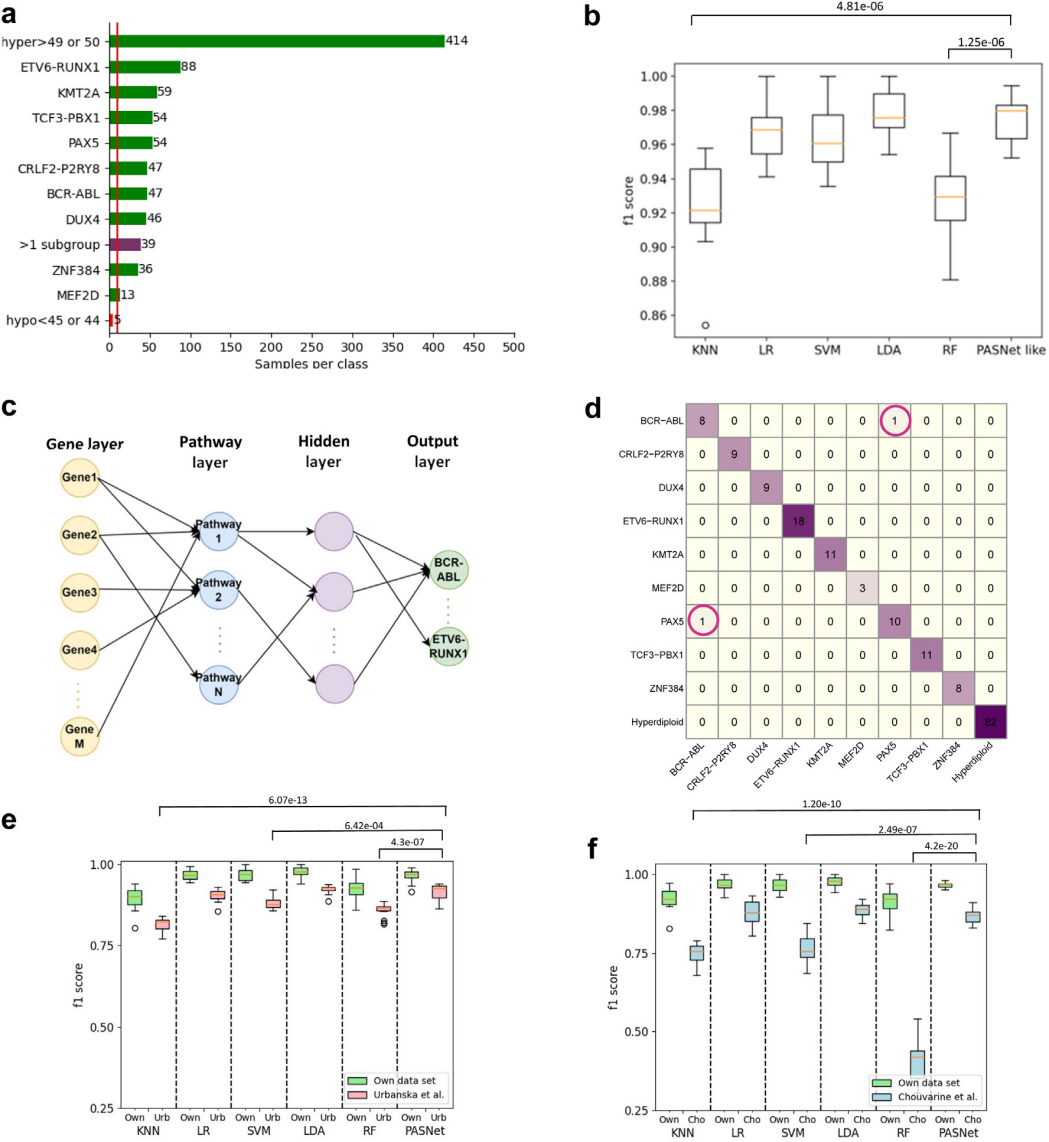
**Machine learning models to predict patients’ subgroups.** (a) The distribution of patients used for ML model development. Here we determined patients’ subgroup (true label) using current diagnostic approaches such as FISH, karyotyping and fusion calling. We included most of them for developing our machine learning models (green bars), with the exception of cases belonging to more than one subgroup and very rare hypodiploid cases (purple bars). (b) A box-whisker-plot for the macro F1 scores of the machine learning models we explored. Orange lines indicate the median of n = 15 experiments. p-values (t-test) with significant difference when comparing with PASNet-like model are indicated on top of the plot. k-nearest neighbour's (KNN), logistic regression (LR), support vector machine (SVM), random forest (RF), linear discriminant analysis (LDA), pathway-associated sparse deep neural network like (PASNet-like). (c) The neural network architecture used in this study. It includes a gene layer as an input layer, a pathway layer, a hidden layer and an output layer. A node in the input layer is only connected to a node in the second layer if this gene exists in the pathway. (d) A confusion matrix from the PASNet-like model. (e–f) Box-whisker-plots showing our model performance in the independent datasets either from Urbanska et al. (e) or from Chouvarine et al. (f). Orange lines indicate the median of n = 15 experiments. p-values (t-test) with significant difference when comparing with PASNet-like model are indicated on top of the plots.

Although paediatric BCP-ALL has a very good overall treatment outcome, the refinement of molecular subtypes allows improved patient stratification towards optimal and individualized treatment.^5^^,^^10^^,^^26^^,^^27^ In contrast to prior studies, our focus was on the integration of molecular subtyping into a diagnostic context through clinALL. For this, we explored various machine learning models to subtype BCP-ALL utilizing gene expression data generated by targeted RNA-seq in routine diagnostics. Fig. 2a illustrates the patients’ subgroup distribution used for model development. We explored five shallow machine learning models and one deep NN model to address this classification problem and incorporated the optimum models into clinALL to give subgroup recommendations to undetermined patients. As shown in Fig. 2b, Supplementary Fig. S2a and Table S1, Linear Discriminant Analysis (LDA) and PASNet-like deep NN models exhibit superior performance compared to other models and achieve macro F1 scores of 97.9% and 97.4% respectively. We call our neural network “PASNet-like” model because it employs a structure similar to PASNet where the neural network is informed with biological gene-pathway information (Fig. 2c).^12^ An advantage of PASNet is that it gives biological interpretations. We carried out further experiments to implement an explainability algorithm DeepSHAP^28^ on our PASNet-like model, and generated summary plots to show the important biological components. Supplementary Fig. S2c are summary plots generated for *CRLF2::P2RY8* and *BCR::ABL1* where important biological components are ranked on the left side. Supplementary Table S2 lists the top ten genes identified in each subgroup. As expected, many of identified genes are in well-known BCP-ALL-associated pathways, such as *PBX1*, *FLT3*, *ABL1* and *BTLA*. Additionally, some genes serve as regulators of these pathways, exemplified by *MDM2* and *MIB1*. It is remarkable that our high accuracy in subgroup prediction was purely based on the gene expression values of targeted RNA-seq. Confusion matrix and error analysis show that we achieve 100% accuracy for the majority of subgroups, whereas that the majority of misclassifications arise from the *BCR::ABL1* or *PAX5* subgroups, which are known to have similar gene expression patterns (Fig. 2d and Supplementary Fig. S2b). We made both LDA and PASNet-like models available in clinALL. Users can choose based on their needs.

To validate our machine learning models, we obtained independent data sets from two previously published paediatric ALL studies, containing targeted RNA sequencing data by Urbanska et al. and the whole transcriptome sequencing data by Chouvarine et al.^17^^,^^18^ We tested all our six models, and the LDA and PASNET-like model perform well on both data sets. The LDA model achieved macro F1 scores of 0.924 and 0.889 in the data from Urbanska and Chouvarine, respectively. The PASNet-like model achieved macro F1 score of 0.915 and 0.864, respectively (Fig. 2e and f, Supplementary Fig. S2a). The drop in F1 score can be attributed partly to the fact that the independent datasets only contain nine (Urbanska data) or seven (Chouvarine data) subgroups of the ten subgroups on which our model was trained. To illustrate, when we train our model on the seven subgroups present in the Chouvarine data, the macro F1 scores for LDA and PASNET-like model are above 0.95 (Supplementary Fig. S2d).

**Fig. 3:**
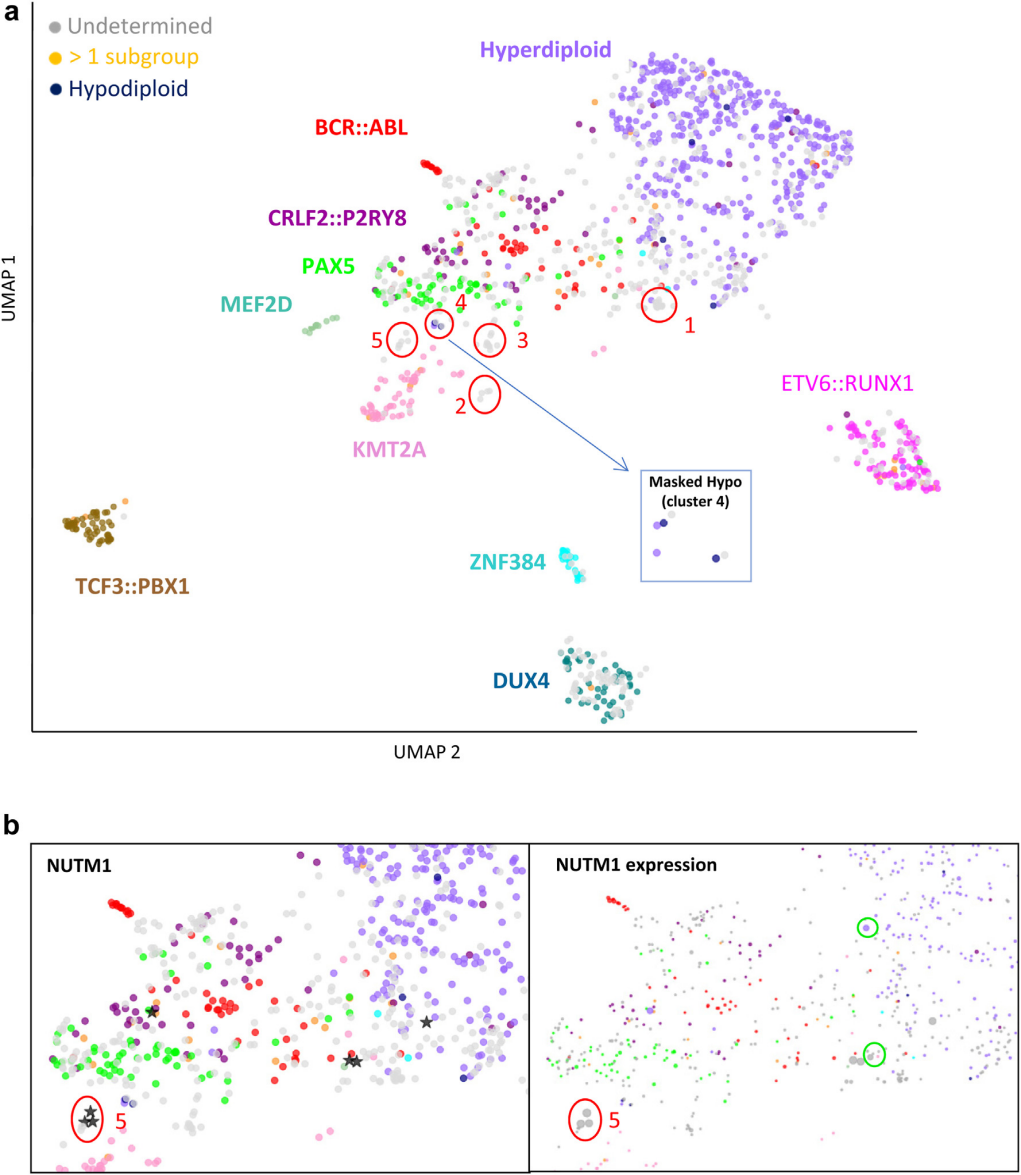
**clinALL confirms and improves the patients’ stratification.** (a) UMAP analysis based on gene expression profile of targeted RNA-seq. While each colour represents a subgroup that is determined by routine diagnostic approaches such as FISH, Karyotyping and fusion calling, the grey dots are the patients that the routine approaches cannot determine the subgroup. Red circle highlights the interesting clusters on which we performed further investigation. Cluster 4 that contains masked hypodiploid cases is zoomed in and shown in the square. (b) clinALL’s function of visualizing individual gene expression helps to confirm patients’ subgroups. Known *NUTM1*-positive cases are marked as stars on the left panel. The expression level of *NUTM1* gene is correlated with the dot size on the right panel. Two additional *NUTM1*-positive cases (green circles) were identified and confirmed by using gene expression function in clinALL.

Our framework provides a fast and robust way of stratifying patients, a key step in optimizing treatment. clinALL subtyping results from UMAP visualization were very consistent with traditional assays. Among the 918 cases on which we could determine the genetic subtypes through conventional molecular cytogenetic and fusion calling, most cases (873/918; 95.1%) successfully clustered based on the genetic subtype indicated by colours. We only detected 45 outliers whose UMAP locations are not near the samples of the same colour. Upon closer examination, we found that some outlier cases were particularly complicated, such as those featuring a complex karyotype or rare fusions, like *JAK2::PAX5*. However, there were still other outlier cases that remained perplexing and lacked a distinct explanation. Recognizing these outliers using clinALL can assist clinicians to differentiate and potentially provide special attention to these cases. In addition to outliers, clinALL also supports the stratification of known subgroups. For example, the detection of hypodiploid BCP-ALL is of high clinical importance, as these cases have a poor prognosis and require an intensive treatment regimen. With clinALL, we were able to identify “masked hypodiploid” cases, a special type of hypodiploid cases often mistaken as hyperdiploid.^29^ In our cohort, hypodiploid cases were rare and located either in cluster 4 or scattered among the hyperdiploid cases (Fig. 3a). Notably, we observed two hyperdiploid cases (purple) in cluster 4 next to the hypodiploid cases (dark blue). To investigate this further, we performed SNP array analysis, which revealed that these cases were actually masked hypodiploids. Another example comes from the recognition of *NUTM1* cases by clinALL. *NUTM1*-positive ALL is defined by the fusion between *NUTM1* and one of the partner genes, which normally leads to high expression of *NUTM1* gene. With the gene expression function in clinALL, we discovered two more cases with prominent *NUTM1* gene expression which were not detected by fusion-calling (green circles in the right panel of Fig. 3b). Upon a closer examination, we found that these two cases did in fact contain reads supporting the presence of a *NUTM1* fusion transcript, but were missed due to a low read count below our bioinformatic pipeline threshold. Conversely, cluster 5 contained cases with low expression of *NUTM1* gene despite their proximity to true *NUTM1*-positive cases. The gene expression function in clinALL gave confidence that these cases were unlikely to be *NUTM1*-positive.

clinALL can also give subgroup recommendations to patients that we could not stratify using routine diagnostic approaches. In our cohort, we could not determine the genetic subgroups in 33% (447/1365) of the cases (grey in Fig. 3a). They include B-others, various “like” subgroups (e.g., *BCR::ABL1*-like and *ETV6::RUNX1*-like), and cases that require additional methods, but are currently not included in the diagnostic routine, such as the *DUX4*-positive subgroup.^27^ clinALL can give subgroup recommendations to these undetermined cases either by their location on the UMAP or by the machine learning prediction displayed on the web interface. The results from both approaches were mostly in agreement, and the few disagreed cases were mostly due to the high probability threshold we chose (95% probability). Taken together, we could give subgroup recommendations for 78% (347/447) of undetermined cases (Supplementary Table S3), rendering clinALL a valuable addition in the workflow of the routine diagnostics. Among the undetermined cases, we also identified three tight clusters in UMAP (Cluster 1–3 in Fig. 3a). Cluster 1 includes cases with incomplete medical documentation regarding lineage commitment, blast percentage, etc. To identify distinct features in cluster 1, we conducted differential gene expression (DGE) analysis between cluster 1 and the rest of the samples in our cohort. We observed low expression levels of genes such as *CD19, EBF1* and *PAX5* in cluster 1 (Supplementary Fig. S3), indicating that these cases may not be BCP-ALL. This was further confirmed when we subsequently evaluated additional information for these cases, demonstrating the value of clinALL in situations of incomplete medical information. Cluster 2 contained four undetermined cases and one *KMT2A::USP8*-positive case. This UMAP location prompted us to further examine these four undetermined cases and we found that all of them contained *KMT2A::USP2* fusions. They were not detected by fusion-calling and FISH, as the read count was below the threshold, and routinely used FISH probes cannot reliably detect rearrangements resulting from small inversions, deletions or insertions.^30^ This underscores the importance of clinALL in cases where standard assays fail. Currently, the AIEOP-BFM, EsPhALL, Interfant and ALLTogether protocols do not take into account single nucleotide variants (SNVs) for risk stratification. Consequently, diagnostic laboratories do not routinely examine SNVs. Based on UMAP location, we hypothesized that cluster 3 could be composed of cases bearing a *PAX5* p.P80R mutation,^26^ which was later confirmed by mutation calling and Sanger sequencing. Therefore, the UMAP in clinALL can also aid in identifying subgroups with important point mutations.

**Fig. 4:**
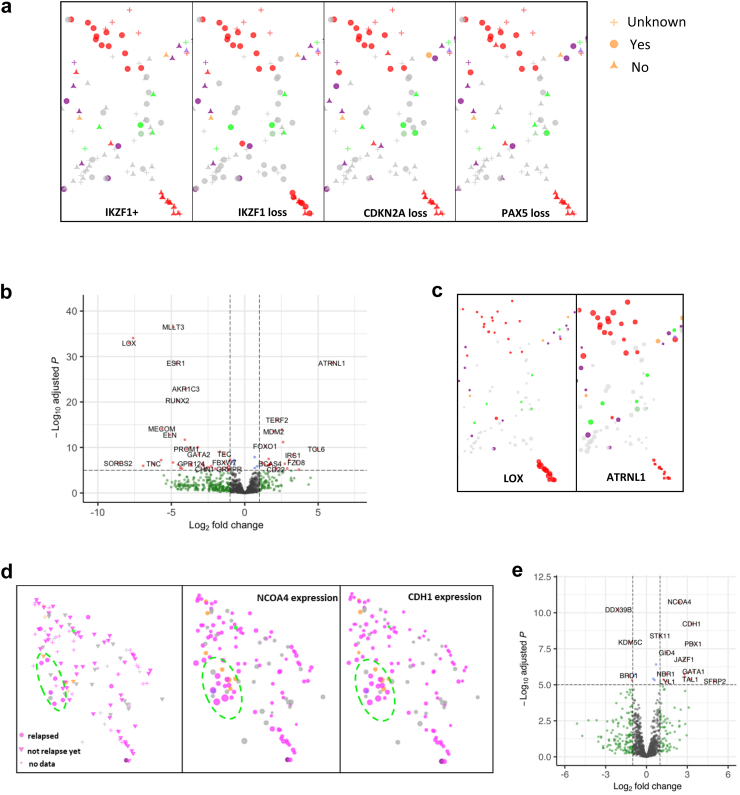
**clinALL identifies subpopulations within subgroups and uncovers clinically relevant molecular insights.** (a) Two of the subpopulations of *BCR::ABL1* are entirely dependent on *IKZF1*^plus^ status (the most left panel), but only partially associated with the loss of *IKZF1, CDKN2A* and *PAX5* (2nd, 3rd, 4th panel). (b) Volcano plot of the differential gene expression analysis on the two subpopulations of *BCR::ABL1*. Genes such as *ATRNL1* and *TERF2* were highly expressed in *IKZF1*^plus^-positive cases, while *LOX, MLLT3, ZFPM2,* and *RUNX2* were down-regulated. (c) Visualization of the top differentially expressed genes by clinALL gene expression function. (d) A subpopulation (green circle) located on the left side of the *ETV6::RUNX1* subgroup exhibits high frequency of relapse. (e) Volcano plot of the differential gene expression analysis comparing *ETV6::RUNX1* cases that are either inside or outside the green circle.

clinALL is a diagnostic framework that brings together diverse clinical and genomic information through a graphical gene expression profile. Through the application of clinALL, we were able not only to identify subpopulations within subgroups, but also to gain insights into the clinical and genetic features that distinguish these subpopulations. We observed that the *BCR::ABL1*-positive subgroup had three major subpopulations (Supplementary Fig. S4a), and two of them were linked to a different *IKZF1*^plus^ status (Fig. 4a), a type of second order genomic information defined by the loss of *IKZF1* gene along with the loss of one of the four other genes (*CDKN2A/B, PAX5, ETV6*), or pseudoautosomal region 1 (PAR1), but not *ERG* loss.^31^ These two subpopulations were entirely dependent on *IKZF1*^plus^ status but were only partially associated with the loss of *IKZF1, CDKN2A* and *PAX5*. To gain more insight, we performed DGE analysis and identified *ATRNL1* and *TERF2* as highly expressed in *IKZF1*^plus^ cases while *LOX, MLLT3,* and *RUNX2* were down-regulated (Fig. 4b). This was further confirmed by the gene expression function in clinALL, where the expression level of *LOX* and *ATRNL1* can be visualized (Fig. 4c). Similarly, we observed two subpopulations of *DUX4*-positive cases that were dependent on *ERG* deletion status, indicating a distinct global expression pattern in *ERG*-positive and *ERG*-negative samples with *DUX4* rearrangements (Supplementary Fig. S4b and c). Subpopulations in *BCR::ABL1*/*BCR::ABL1*-like and *KMT2A* were also observed, both of which correlate with a loss of *PAX5* (Supplementary Fig. S4d and e). Another exciting discovery by clinALL is that we can identify relapse-prone subpopulation within *ETV6::RUNX1* subgroup after integrating clinical information with gene expression patterns. Predicting cancer relapse is one of the most important questions in cancer management. Typically, gene expression patterns from diagnostic samples are not expected to be indicative of cancer relapse. However, by visualizing relapse information on top of gene expression patterns, we observed that seven out of ten relapsed cases within the *ETV6::RUNX1* subgroup were clustered together (Fig. 4d). This unexpected finding suggests the presence of biomarkers indicating elevated relapse risk even at the time of diagnosis. To gain further insights into the underlying mechanisms, we conducted DGE analysis and found that genes such as *NCOA4* and *CDH1* were highly expressed, while *DDX39B* demonstrated low-level expression (Fig. 4d and e). We also carried out reactome pathway analysis to identify pathways involved in subpopulations, and results are shown in Supplementary Table S4.

## Discussion

clinALL is a fast and cost-effective data integration and analysis framework for supporting clinical decision-making and translational research. It does not require additional data or curation beyond what is already generated by the diagnostic protocol. Most importantly, it makes second order information, usually overlooked in routine diagnostics, easily accessible through its intuitive interface and compelling visualizations. Clinicians and researchers can associate all the data in one place, rather than navigating through various tables and databases. This greatly facilitates clinical decision-making and the discovery of patterns in patient cohorts. To the best of our knowledge, there is currently no tool like clinALL that can not only integrate both clinical and genomic data, but also provide machine learning/neural network recommendations, integrating everything into routine diagnostics (Supplementary Table S5). While generic oncological framework like MOALmanac and OncoKB,^1^^,^^4^ along with gene expression visualization tools such as Seurat,^32^ and BCP-ALL classifiers like ALL-Sorts and ALLCatchR,^10^^,^^33^ provide some functionality that clinALL achieves, none of them offers the comprehensive integration that clinALL delivers for routine diagnostics. Moreover, clinALL is ready to be used for many types of hematologic neoplasia and can provide an analysis output within an hour after the diagnostic results are uploaded. It is easily transferable, making it especially valuable for small to medium-sized clinics in developing countries.

We decided to use BCP-ALL as a use case because currently a multitude of methods are used in parallel to generate large amount of genetic and clinical data, yet there lacks an efficient data integration platform. By integrating various data sources from BCP-ALL, clinALL has demonstrated great potential to facilitate translational research. Our finding of subpopulations of *BCR::ABL1* that are dependent on *IKZF1*^plus^ status may have implications for future treatment plans, as *IKZF1*^plus^ cases are associated with dismal survival. We also discovered a subpopulation of *ETV6::RUNX1* cases with a high frequency of relapse. *ETV6::RUNX1* patients are usually stratified into the non-high risk treatment arms (standard or intermediate risk) and receive less intensive treatment. Of importance, about half of paediatric BCP-ALL relapses are initially diagnosed as non-high risk,^33^ indicating the need to improve risk stratification for these patients. clinALL enables potentially further improvement of stratification for *ETV6::RUNX1* patients and pinpoints those at risk of relapse. Further assessment of potential biomarkers such as *NCOA4* and *CDH1* may help to translate clinALL findings into clinical decision-making strategies and improve treatment outcomes.

clinALL is a clinical framework that integrates neural networks into routine diagnostic analyses of cancer genomic data. We explored a range of machine learning models and developed classification models to predict patient subgroups based on gene expression data. Our models are verified not over fitting by learning curves (Supplementary Fig. S2e and f). We did not introduce manual feature selection or other data types such as mutation or clinical data because our model already achieved a very good performance for the task of predicting subgroups. As shown in Fig. 2d and Supplementary Fig. S2b, 100% accuracy were achieved for most of the subgroups, and only one misclassified case were reported in *BCR::ABL1* and *PAX5* subgroups. While incorporating other data types or manual feature selection may show significant improvement in more challenging tasks such as predicting survival, it necessitates careful experimental design to mitigate potential biases toward pre-existing knowledge. It is important to consider that our F1 score of 97% only reflects the model’s ability to predict cases within the ten subgroups which were used for training. For the smaller subgroups with insufficient training samples, or B-other cases which do not belong to any subgroup, our model only provides a heuristic on which of the ten subgroups are closest to the one under investigation.

Targeted RNA-seq rather than whole transcriptome sequencing is used in many diagnostic settings due to limited budgets and bioinformatics supports in many clinical and diagnostics laboratories.^19^ Our results demonstrated that targeted RNA-seq can reach conclusions highly comparable to whole transcriptome analysis as the major subgroups identified here are extremely consistent with previous publications.^21^^,^^31^ In very few scenarios where the gene of interest has no probe or the probe in the targeted panel performs poorly, as observed with the *DUX4*–positive cases, we overcame this limitation by analysing global gene expression patterns using clinALL. If some of the traditional diagnostic assays may be replaced by Next Generation Sequencing (NGS) in the future, targeted RNA-seq in combination with an easily transferable analytical framework such as clinALL will be a viable option for many laboratories, since whole transcriptome sequencing requires significantly more data storage and bioinformatics support.

In summary, clinALL represents an approach for data integration, visualization and analysis in a diagnostic setting. In contrast to previous clinical frameworks, clinALL includes both second order genetic information and a variety of clinical data. By using BCP-ALL as a use case, we showed that this approach enables us to take full advantage of the available diagnostic data, especially NGS data. Creating similar frameworks for other disease entities promises to enable the efficient and equitable delivery of personalized medicine not only in large comprehensive cancer centres, but also in less equipped small clinics. Finally, the approach also has high potential for cross-entity analysis, expanding its range of applications and enhancing its utility.

## Contributors

Conceptualization and study design: MT and AKB; Bioinformatics analysis: MT, AE and WN.; Data curation: AE, MT, ZA, SP, AKB, MZ, KM, AP; Patients data selection and preparation: SP, CS, GE, MH, MM, MS (Martin Schrappe), KM, AP, GC, MS (Martin Stanulla) and JL; Diagnostic validation: JL, SP, AKB; Statistical analysis: MT and MZ; Software and ML development: MT, AE and PF; Resources, manuscript review and discussion about study: WN, BS, MS (Martin Schrappe), GC, BYR and MS (Martin Stanulla), AvB, JMM; Data analysis: MT, ZA and AKB; Original draft: MT, ZA and AKB; Funding acquisition: AKB, TI, WN. Data verification: MT, ZA, MS (Martin Stanulla) and AKB. All authors have read and agreed on the final version of the manuscript.

## Data sharing statement

The data presented in this study are available on request from the clinical trial groups. The code for clinALL and machine learning models are available in the following Git repository: http://git.l3s.uni-hannover.de/tang/clinALL.

## Declaration of interests

GC has received research support from German Cancer aid (70112958) and German Research Society (KFO 5010/1). GC has also participated in an Advisory Board, and received consultancy fees from JazzPharma, and honoraria from Amgen.
