# Supplementary material for: An artificial intelligence-assisted clinical framework to facilitate diagnostics and translational discovery in hematologic neoplasia
Source: eBioMedicine. 2024 May 28;104:105171. doi: 10.1016/j.ebiom.2024.105171 (PMC11154115; doi:10.1016/j.ebiom.2024.105171)
Supplement: Supplementary Figs. S1–S3 and Tables S1–S4 [file mmc1.docx]

**Supplementary Figures**

**
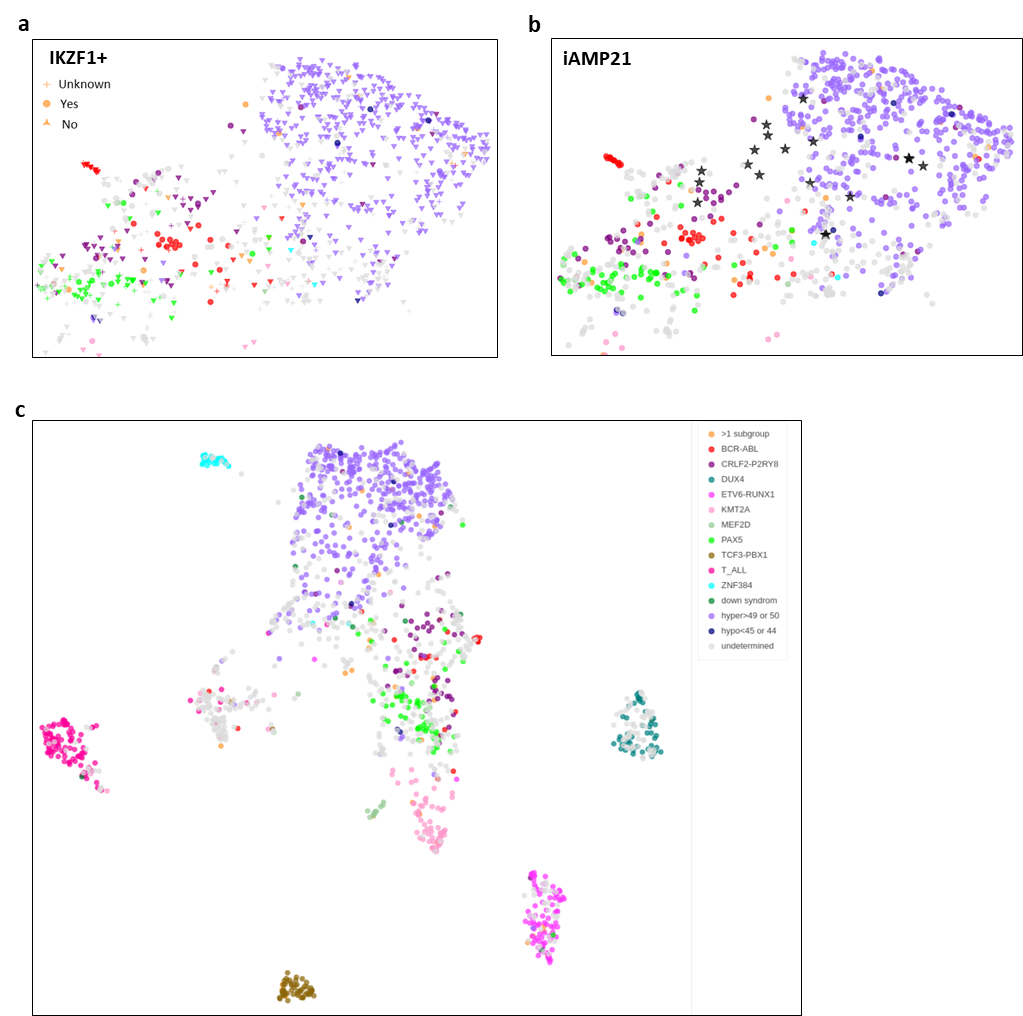
**

**Supplementary Figure 1. clinALL, a clinical framework that incorporate second order genetic information and clinical information for hematologic neoplasia.** (a) A screen shot of clinALL where *IKZF1*^+^ status, a second order genetic information, was displayed on top of UMAP. (b) A screen shot of clinALL where iAMP21 cases are highlighted with stars. (c) A UMAP including the T-ALL samples in addition to the BCP-ALL

**
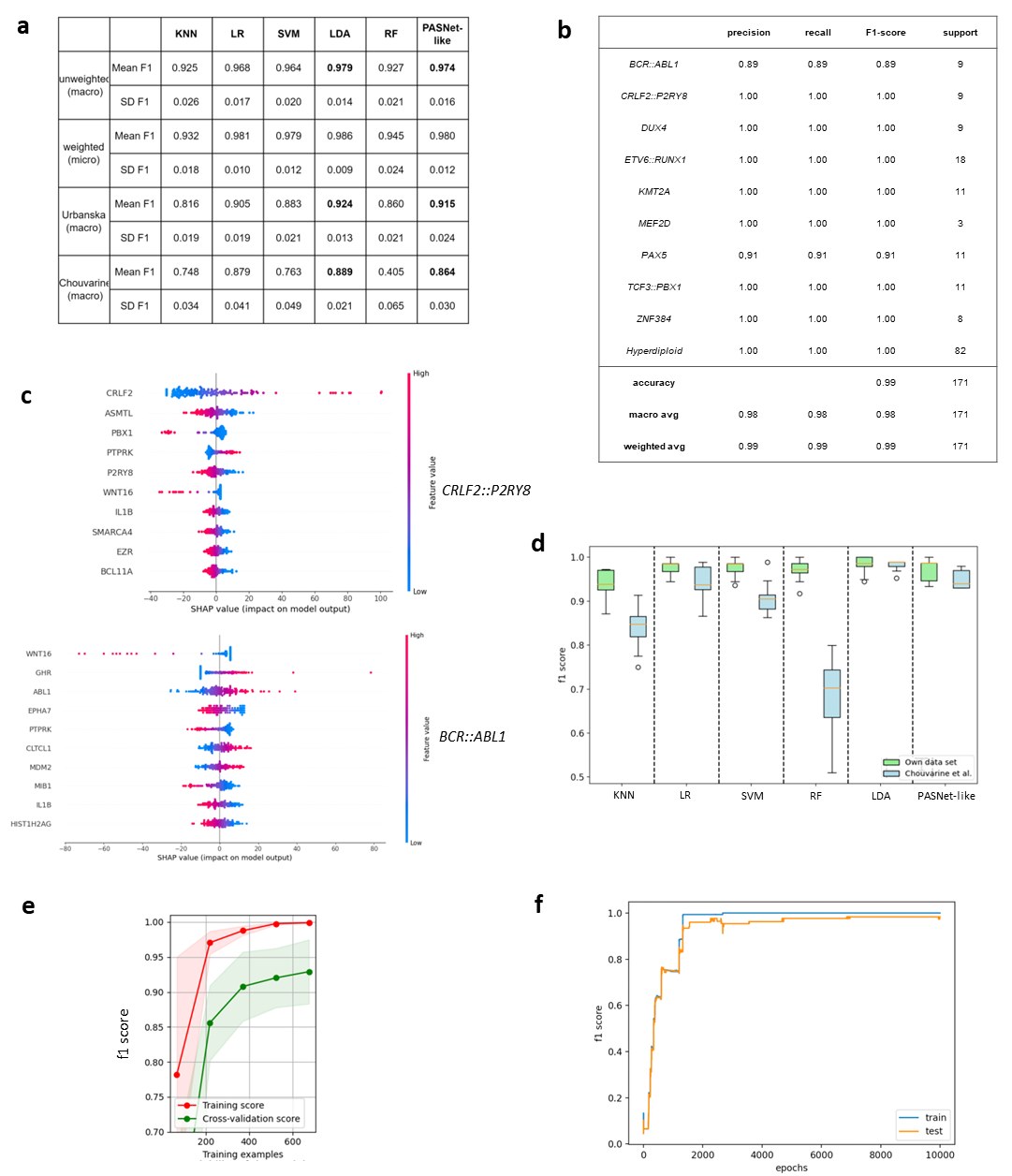
**

**Supplementary Figure 2. Machine learning models to predict patients‘ subgroups** (a) Top four rows: means and standard deviations (SD) of macro- and micro-F1 scores from our own data. Bottom four rows: mean and SD of macro-F1 scores from two independent datasets. (b) A classification report from PASNet-like model. (c) Summary plots for *CRLF2::P2RY8* and *BCR::ABL1*, generated by our PASNet-like model and DeepSHAP. The top ten important genes in determining the subgroup are listed on the left. Each dot represents one sample. The color indicates the gene expression level and the X-axis shows the SHAP values (feature importance). (d) Macro F1 scores of our models when trained on 7 subgroups that exist in both our own data and data from Chourvarine et al. (e) A learning curve from our LDA model. (f) A learning curve from PASNet-like model.


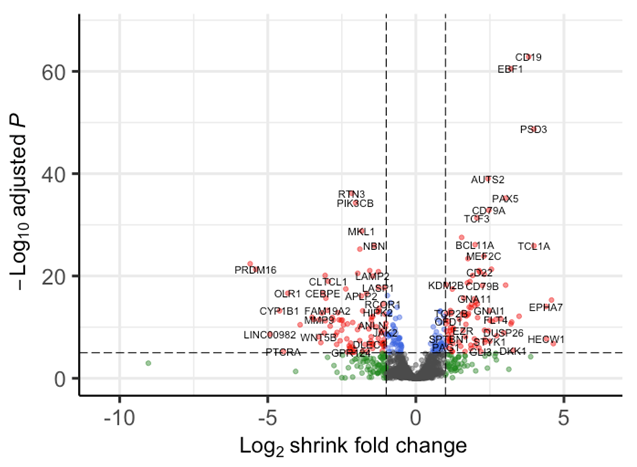


**Supplementary Figure 3. clinALL confirms and improves the patients‘ stratification.** A volcano plot comparing cluster 1 and rest of the samples in our cohort. Differentially expressed genes indicate that cluster 1 contains non-BCP-ALL samples.


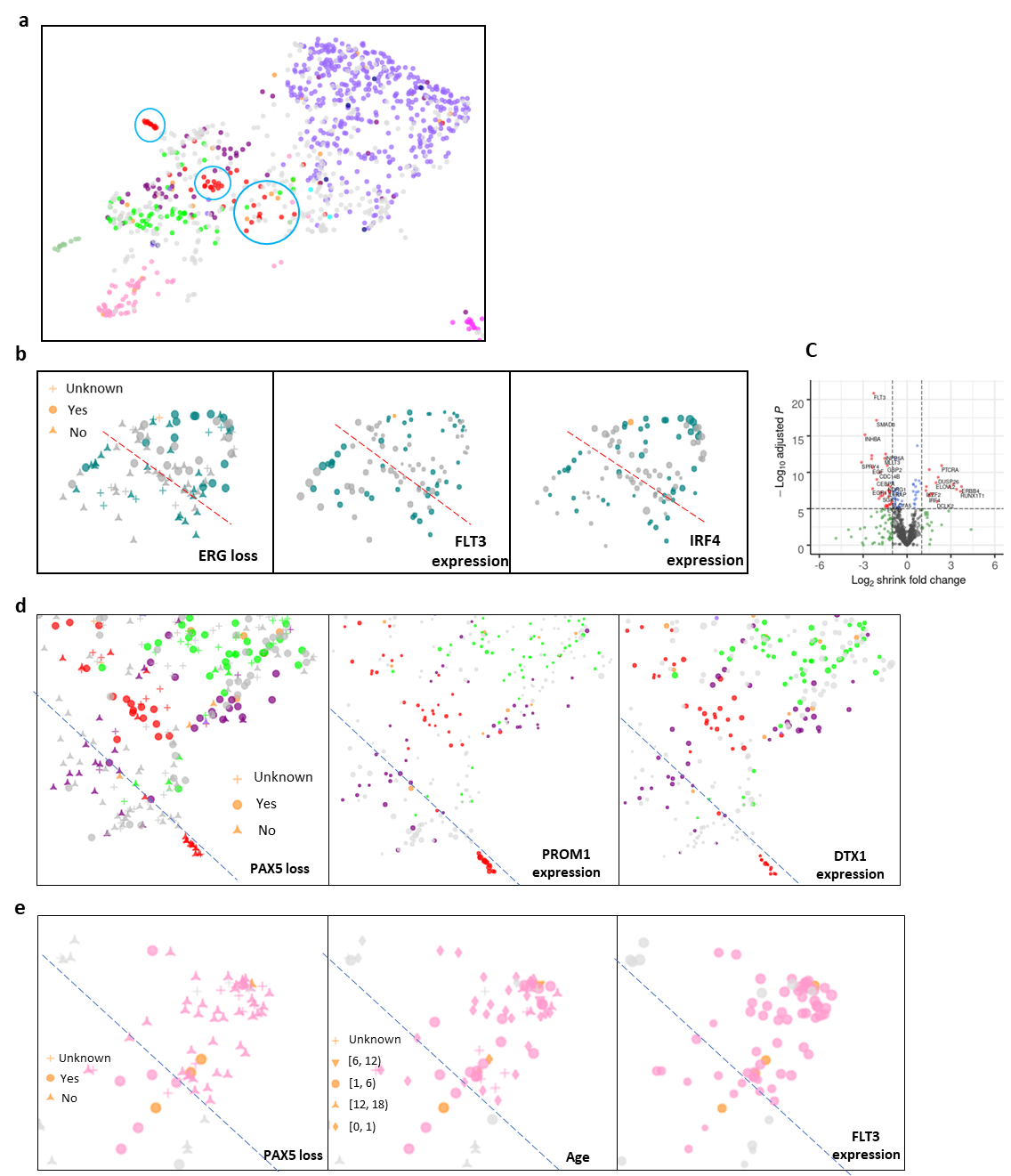


**Supplementary Figure 4. clinALL identifies subpopulations within subgroups and uncovers novel insights.** (a) Three subpopulations of *BCR::ABL1* in our cohort. (b) Two *DUX4*-positive subpopulations which are highly associated with ERG gene loss status. (c) Volcano plot of differential gene expression analysis on the two subpopulations of *DUX4*-positive samples. Genes such as *FLT3, IRF4, SMAD3* and *RUNX1T1* are identified as the most differentially expressed genes. (d) *BCR::ABL1*-like cases exhibit subpopulations that are dependent on *PAX5* gene loss status. (e) Two subpopulations of *KMT2A*-positive samples that are associated with PAX5 gene loss status, as well as correlated with age and *FLT3* gene expression.

**Supplementary Table 1 (Submitted as an excel file): Machine learning model evaluation metrics and hyperparameters.**

**Supplementary Table 2 (Submitted as an excel file): Top ten important genes/nodes in each subgroup.** We implemented an explainability algorithm DeepSHAP on our PASNet-like model and identified genes/nodes that are important for the predictions of each subgroup.

**Supplementary Table 3: Statistics of undetermined cases that the subgroups can be confidently determined by ML or UMAP.** The first column lists the number of cases with the highest prediction probability > 95% in our machine learning model. The second column lists the number of cases determined by location on UMAP. The last column lists the number of cases that can be confirmed by both UMAP and machine learning model (>95% probability).

|  | By Machine Learning  (The highest probability >95%) | | By UMAP location | | Confirmed by both ML and UMAP | |
| --- | --- | --- | --- | --- | --- | --- |
| *DUX4* | | 54 | | 55 | | **54** |
| *ETV6::RUNX1* like | | 27 | | 29 | | **24** |
| *ZNF384* like | | 6 | | 7 | | **6** |
| *KMT2A* like | | 9 | | 2 | | **2** |
| Ph like (*BCR::ABL1, CRLF2::P2RY8, PAX5*) | | 153 | | 130 | | **116** |
| Hyperdiploid | | 180 | | 181 | | **145** |
| Cluster 1 (non B-ALL) | | - | | 16 | | - |
| Cluster 2 (*KMT2A::USP2/8*) | | - | | 4 | | - |
| Cluster 3 (*PAX5* p.P80R) | | - | | 11 | | - |
| Cluster 4 (hypo/masked hypo) | | - | | 6 | | - |
| Cluster 5 (3 NUTM1 + 3 unknown) | | - | | 3 | | - |
| The cases that we can confidently predict: 54+24+6+2+116+145=347  The percentage of undetermined cases that we can confidently predict: 347/447 = **77.6%** | | | | | | |

**Supplementary Table 4 (Submitted as an excel file): Reactome pathway analysis for subpopulations of *BCR::ABL1* and *ETV6::RUNX1*.** For *BCR::ABL1,* enrichment of genes involved in FOXO-mediated transcription was found in the *IKZF1^plus^*-positive subpopulation while genes involved in PI3K/AKT/MAPK signalling were enriched in the *IKZF1^plus^*-negative subpopulation. For *ETV6::RUNX1*, genes involved in cellular senescence, oestrogen receptor and NOTCH signalling were found to be enriched in relapsed subpopulations, and genes involved in ALK signalling, chromatin organization and RNA transcription were found to be enriched in the non-relapsed subpopulation

**Supplementary Table 5: Comparison with other similar tools and platforms.**

|  | **Name** | **Purposes** | **Data types** | **Machine learning/neural networks** | **“Second order” alterations**  (global patterns and interaction between individual aberrations) | **Real time integration into routine diagnostics** | **References** |
| --- | --- | --- | --- | --- | --- | --- | --- |
| **Knowledge base** | MOAlmanac | A clinical interpretation platform to associate genetic alterations with therapeutic opportunities | All cancer types.  Mostly genomic data. | No | Yes. Considered global TMB (tumour mutation burden) | No | 4 |
|  | OncoKB | A precision oncology knowledge base about genetic alterations and clinical actionability | All cancer types Mostly genomic data. | No | No | No | 1 |
| **Tools for visualizing gene expression** | Seurat | An R package to analyse gene expression especially single cell transcriptomic measurements with the possibility of integrating couple more data types. | Gene expression data, especially single cell RNAseq data. | No | Yes. One can visualize global gene expression patterns. | No | 32 |
| **BCP-ALL specific machine learning classifiers** | ALLsorts | A BCP-ALL subtype classifier | RNA-seq data from B-ALL | Yes.  Only shallow machine learning | Yes. Used global expression pattern to predict subtypes, but the output does not contain visualization of global expression pattern. | No | 10 |
|  | ALLCatchR | A BCP-ALL subtype classifier | RNA-seq data from B-ALL | Yes.  Only shallow machine learning. | Yes. Used global expression pattern to predict subtypes, but the output does not contain visualization of global expression pattern. | No | 33 |
| **Genomic and clinical data integration in a diagnostic setting** | clinALL | A clinical data integration and visualization platform for routine diagnostics. Suitable for any clinics that generate gene expression data. | Gene expression data, genomic data as well as clinical data such as MRD, drug response, sex etc. | Yes.  Both shallow machine learning and neural networks. | Yes | Yes.  As soon as new patient data arrives, one can visualized their location on UMAP and receive subgroup recommendations within an hour |  |
